# Whole‐genome sequencing revealed a novel long‐range deletion mutation spanning *GNAS* in familial pseudohypoparathyroidism

**DOI:** 10.1002/mgg3.2144

**Published:** 2023-01-20

**Authors:** Yangfan Fei, Lv Liu, Lixia Wu, Ou Wang, Xiaoping Xing, Aiping Li, Lingyi Huang

**Affiliations:** ^1^ Department of Endocrinology and Metabolism Meishan Municipal People's Hospital Sichuan China; ^2^ Department of Endocrinology, Key Laboratory of Endocrinology, National Health Commission Peking Union Medical College Hospital, Chinese Academy of Medical Science & Peking Union Medical College Beijing China

**Keywords:** 20q13.2, GNAS, Pseudohypoparathyroidism, Pseudo‐pseudohypoparathyroidism, whole‐genome sequencing

## Abstract

**Background:**

Pseudohypoparathyroidism (PHP) is a series of diseases related to pathological changes and neurocognitive and endocrine abnormalities, mainly due to the *GNAS* mutation on chromosome 20q13.2, which weakens receptor‐mediated hormone signal transduction. Considering its complex genetic and epigenetic characteristics, *GNAS* may produce complex clinical phenotypes in families or sporadic cases. This study presented a case of familial PHP caused by a deletion mutation in the 20q13.2 region.

**Methods and Results:**

The proband and her second daughter had PHP, and the proband's mother had pseudo‐PHP. Whole‐genome sequencing revealed that the proband had an 849.81 kb deletion spanning *GNAS* near the maternal 20q13.2 chromosome. Multiplex ligation‐dependent probe amplification methylation analysis indicated that the proband as well as her mother and second daughter had seemingly abnormal *GNAS* methylation. This is different from the phenotype (feeding difficulty, slow growth, and special facial features) of previously reported cases with the deletion of fragments near the 20q13.2 chromosome.

**Conclusions:**

This report demonstrated the variability of 20q13.2 deletion phenotypes and the clinical importance of using multiple molecular genetic detection methods.

## Graphical abstract

Pseudohypoparathyroidism (PHP) and related diseases are associated with a series of pathological changes, neurocognitive, and endocrine abnormalities, mainly due to the GNAS mutation on chromosome 20q13.2, which leads to the weakening of receptor‐mediated hormone signal transduction. Because GNAS has complex genetic and epigenetic characteristics, it may produce more complex clinical phenotypes of family or sporadic cases. Here, we introduce family cases of three generations in China. The proband and her youngest daughter showed moon‐face, short stature, hormone resistance, and Albright hereditary osteodystrophy. The mother of the proband showed pseudo‐pseudohypoparathyroidism phenotype. This is different from the phenotype (feeding difficulty, slow growth, and special facial features) of the previously reported cases with deletion of fragments near 20q13.2.

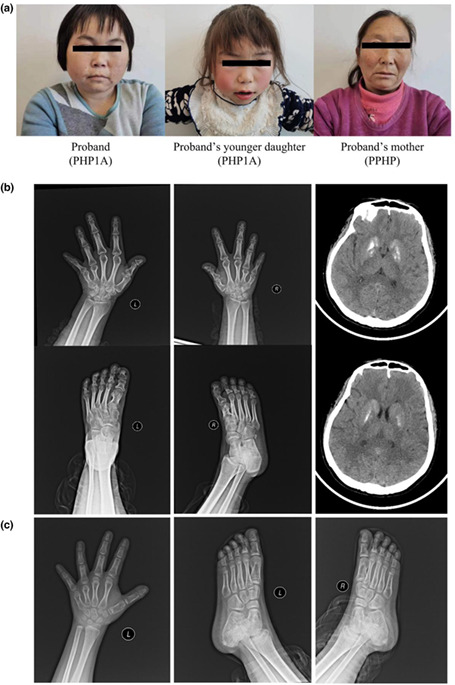

## 1 INTRODUCTION

Pseudohypoparathyroidism (PHP) and pseudo‐pseudohypoparathyroidism (PPHP) are endocrine system diseases caused by genetic and/or epigenetic changes in the parathyroid hormone/parathyroid hormone‐related protein (PTH/PTHrP) signaling pathway involving the alpha subunit of the stimulatory G protein (Gsa) (Jüppner, 2021). PHP is a rare disease characterized by high heterogeneity and serious damage to multiple systems of the body. Its clinical manifestations include brachydactyly, short stature, stocky body build, early‐onset obesity, and heterotopic ossification related to endocrine abnormalities (Thiele et al., 2016). The occurrence of PHP is mainly related to mutations in *GNAS*, which encodes Gsa. *GNAS* is a complex imprinted gene locus with multiple transcription units (Jüppner, 2021). In addition to the 13 exons encoding Gsa, several gene products with physiological effects can be transcribed using additional first exons, including GNAS A/B, GNAS‐XLas, and GNAS‐NESP55 sites, upstream of the Gsa exons (Levine, 2012; Mantovani et al., 2010). In some specific tissues, such as the thyroid, gonad, and pituitary gland, *GNAS* is expressed as a maternal gene, while the paternal allele expression is inhibited (Jüppner, 2021; Thiele et al., 2016). Maternal genetic mutations cause Albright hereditary osteodystrophy (AHO) and resistance to some hormones, such as PTH, thyroid‐stimulating hormone (TSH), gonadotropin‐releasing hormone, and growth hormone‐releasing hormone (Mantovani et al., 2018). Paternal inactivation mutations usually only cause AHO, with no severe resistance to PTH and other hormones (Lemos & Thakker, 2015; Turan et al., 2014).

*GNAS* mutations have been reported in several cohorts and cases. The common types of mutations include frameshift mutations, which can lead to loss of function and missense mutations (Snanoudj et al., 2020). Deletion mutations comprising the 20q13.32 region of *GNAS* are rare; to date, only eight patients with such mutations have been reported in six studies (including de novo, paternal, and maternal mutations). The phenotypic features of these patients are mainly characterized by feeding difficulties, slow growth, abnormal facial appearance, and mental retardation, with some patients showing an incomplete AHO phenotype (Aldred et al., 2002; Balasubramanian et al., 2015; Butler et al., 2013; Geneviève et al., 2005; Liu et al., 2022; Solomon et al., 2011). This may be different from the typical PHP phenotype that includes PTH and TSH resistance, short bones, short stature, and stocky build (Mantovani et al., 2018).

The 20q13.32 fragment contains multiple pathological genes, including *GNAS*, with complex genetic and epigenetic characteristics; hence, it may produce phenotypic features that are difficult to diagnose. Here, we present a clinical report of patients with a family history of PHP in China and have performed genetic testing on three generations of the family. Whole‐genome sequencing (WGS) revealed that 20q13.2 had a deletion of 849.81 kb, and methylation‐specific multiplex ligation‐dependent probe amplification (MS‐MLPA) results indicated that the heterozygous deletion of the maternal *GNAS* complex regulatory element led to abnormal methylation in this region. This study contributes to the clinical understanding of PHP diagnosis and genetic detection strategies.

## 2 MATERIALS AND METHODS

### 2.1 Editorial policies and ethical considerations

This study was approved by the ethics committee of the Meishan Municipal People's Hospital, and informed consent was obtained from the patient and her parents for genetic testing. The clinical data and laboratory examination results of the patients were collected.

### 2.2 Genomic DNA extraction

Ethylenediaminetetraacetic acid anticoagulant tubes were used to collect the peripheral blood of the proband and family members (her husband, mother, and two daughters), and a Blood Genomic DNA Kit (Tiangen Biochemical Technology Co., Ltd., Beijing, China) was used to extract genomic DNA, according to the manufacturer's instructions.

### 2.3 MS‐MLPA

The genomes of the proband and her family members (her husband, mother, and two daughters) were tested for MS‐MLPA. Multiple connected probes were amplified using the SALSA MS‐MLPA Probemix ME031‐B2 kit (MRC Holland, Amsterdam, Netherlands) in accordance with the manufacturer's instructions. DNA samples from healthy individuals were used as controls to detect PHP‐related copy number variations (CNVs) and methylation abnormalities.

### 2.4 Proband WGS analysis

The proband genome was prepared using the Hieff NGS® OnePot DNA Library Prep Kit for Illumina® kit (Yeasen Biotechnology Co., Ltd., Shanghai, China) according to the manufacturer's instructions. The DNA library was sequenced with high throughput in the PE100 mode on the DNBSEQ‐T7 platform (BGI Co., Ltd., Shenzhen, China). The standard process of bioinformatic filtering was improved according to the method described in a previous study (Kohda et al., 2016). The FASTQC software was used to control the quality of the original data. After removing low‐quality reads, BWA software was used to compare the sequence with the reference genome (GRCh37/hg19). The database of the normal population (dbSNP, www.ncbi.nlm.nih.gov/snp/; ExAC, www.exac.broadinstitute.org/; and 1000 Genomes, www.1000genomes.org/) was checked for the screened mutations, and hazard prediction analysis was conducted on the reliable mutation spectrum after filtering the invalid mutations (SIFT, www.sift.bi.a‐star.edu.sg/; PolyPhen‐2, www.genetics.bwh.harvard.edu/pph2/; and MutationTaster, www.mutationtaster.org/). The pathogenicity of the mutation was rated using ACMG (Richards et al., 2015).

In addition, CNVs with an interval size of ≥100 kb were detected from offline data; genes contained in CNVs were analyzed and microdeletions/duplications in the sample data were judged. Finally, CNVs were annotated using the Decipher (www.decipher.sanger.ac.uk/), dbVar (www.bigd.big.ac.cn/databasecommons/), DGV (www.dgv.tcag.ca/dgv/app/home/), ClinGen (www.clinicalgenome.org/), and OMIM (www.omim.org/) databases. Sequencing was performed by Shanghai Wehealth Biotechnology Co., Ltd. (Shanghai, China).

## 3 RESULTS

### 3.1 Case description

**FIGURE 1:**
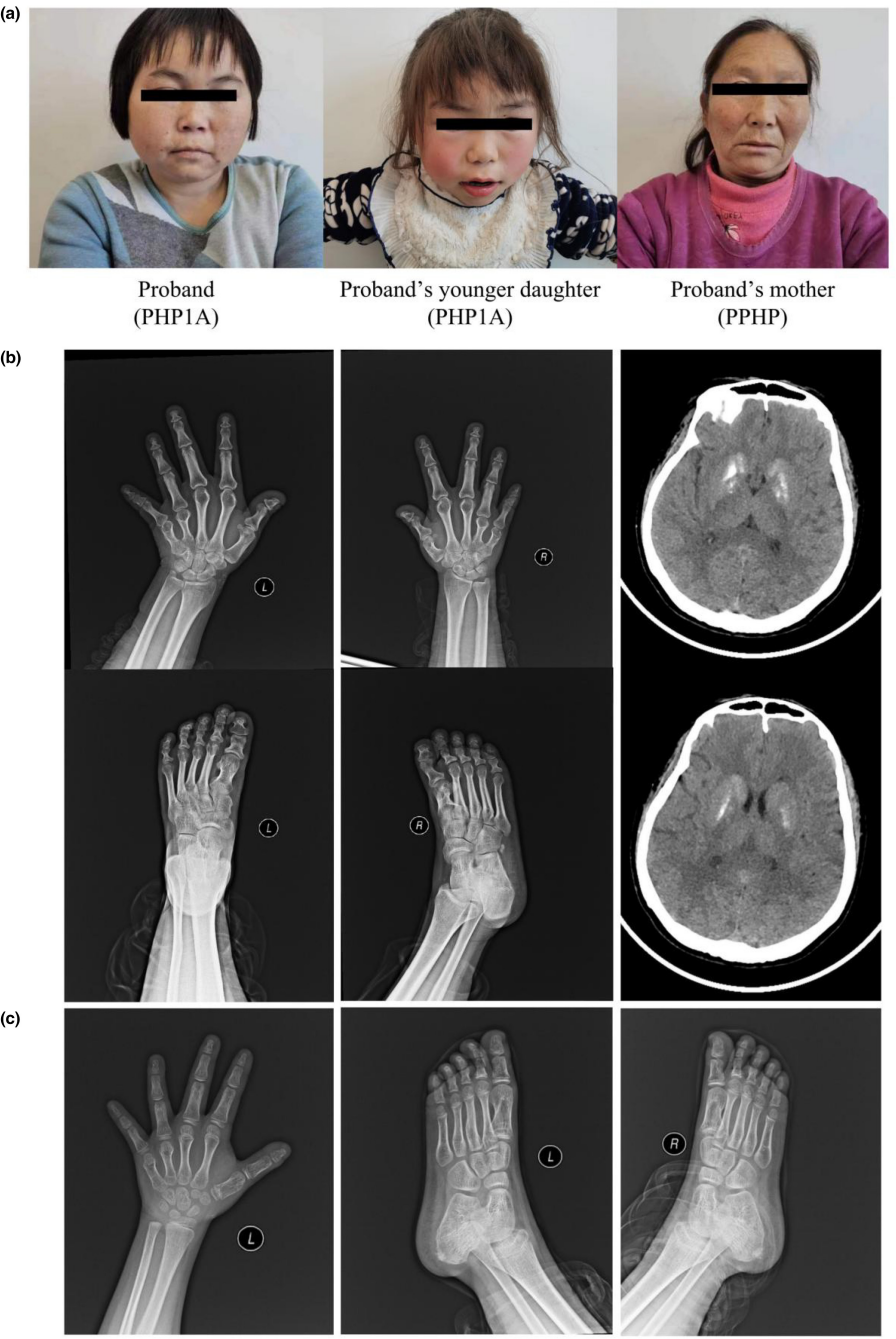
Images and data of the family with PHP. (a) The family comprises a 33‐year‐old proband, her mother, husband, and two daughters. The proband shows features such as round face, obesity, short stature, and short fingers. Her younger daughter also shows round face and short stature. The proband's mother has no similar facial or physical features. (b) X‐ray examination shows that the proband's wrists and palms are completely closed, and the 1st, 4th, and 5th metacarpal bones, 1st–5th distal phalanges, 5th middle phalanx, 5th distal phalanx, and thumb phalanges are short and thick. The epiphysis of both feet is completely closed; the first metatarsal, proximal metatarsal, 2nd–5th proximal metatarsal, middle metatarsal, and distal phalanges of both feet are thick and short; and some joint spaces are narrow. Head CT shows scattered bilateral frontal and parietal lobes with nodular high‐density shadows and bilateral basal segmental high‐density shadows. (c) X‐ray examination shows that the ossification center of the left ulna and styloid process of the ulna do not appear in the proband's younger daughter. The ossification center of the distal radius appears, and the epiphyseal line is not closed. Seven ossification centers are present in the wrist and dense white lines are present in the scaphoid, with no lenticular bone. Metacarpophalangeal epiphysis is partially closed, and the sesamoid bone in the medial thumb does not appear. The epiphysis of both feet is partially healed, and no definite abnormal bone changes are found.

**FIGURE 2:**
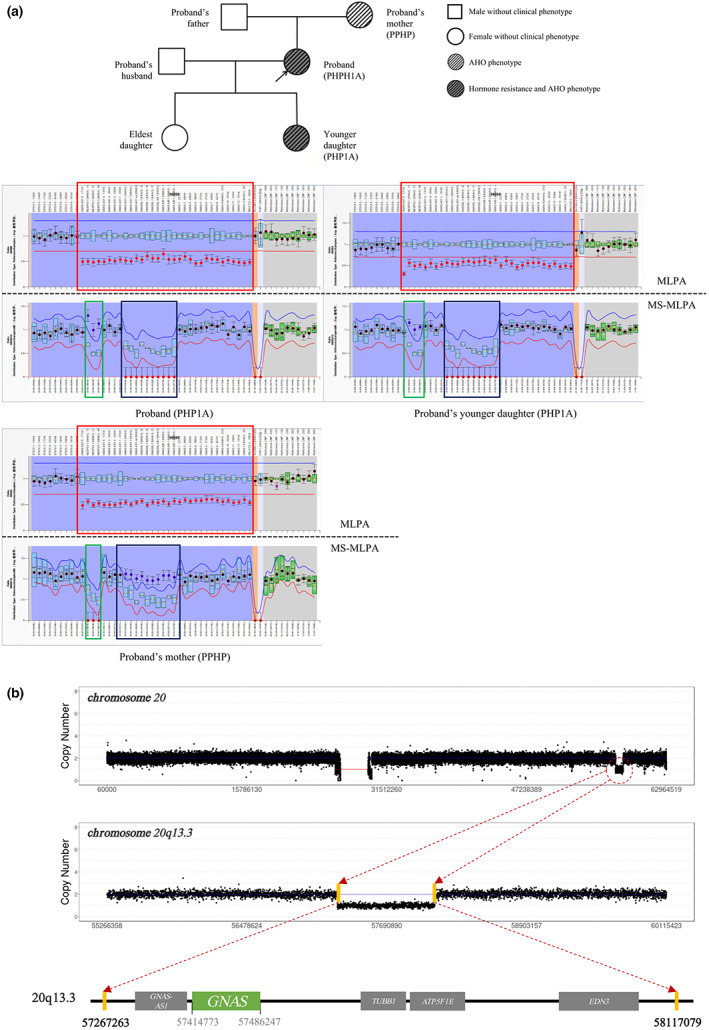
Results of genetic testing. (a) MS‐MLPA results of the family indicate a *GNAS‐AS1/GNAS* region copy number heterozygous deletion (shown in the red box) in the proband, her younger daughter, and mother, whereas no abnormality is observed in her husband and older daughter. Methylation sensitivity test results suggest that the proband, her younger daughter, and her mother have abnormalities. The proband and her younger daughter have *GNAS‐AS1:TSS‐DMR*, *GNAS‐XL:Ex1‐DMR*, and *GNAS A/B:TSS‐DMR* maternal loss‐of‐methylation (shown in the blue box) with a *GNAS‐NESP:TSS‐DMR* gain‐of‐methylation (shown in the green box). However, the proband's mother has *GNAS‐AS1:TSS‐DMR*, *GNAS‐XL:Ex1‐DMR*, and *GNAS A/B:TSS‐DMR* gain‐of‐methylation (shown in the blue box) with *GNAS‐NESP:TSS‐DMR* loss‐of‐methylation (shown in the green box), which may be attributed to paternal inheritance. (b) Whole‐genome sequencing (WGS) results suggest that the proband has an 849.81 kb deletion near 20q13.32, seq[GRCh37]del(20)(q13.32) chr20:g.57267263_58117079del. This segment spans the following five pathological genes included in the OMIM database: *GNAS‐AS1*, *GNAS*, *TUBB1*, *ATP5F1E*, and *EDN3*.

The patient was a 33‐year‐old woman with two daughters. From the age of 20 years, she experienced unexplained numbness of the limbs, stiffness of the hands, and fatigue, but without muscle pain or twitching. The above symptoms recurred 10 days before admission and were accompanied by dizziness and visual rotation. Physical examination upon admission revealed mild anemia, obesity, round face, short neck, short finger (toe) deformity (Figure 1), and a shielded chest. The patient's height, weight, and BMI were 132 cm, 43.9 kg, and 25.19 kg/m^2^, respectively. Laboratory examinations revealed that the patient's blood calcium was slightly lower than normal, and the parathyroid hormone level was elevated (Table 1). Cranial computed tomography (CT) showed scattered nodular high‐density shadows in the bilateral frontal and parietal lobes. Radiographic examination of the bone age showed that the left carpometacarpal and bipedal epiphysis were completely closed. Bilateral 1st, 4th, and 5th metacarpals, 1st–5th distal phalanges, 5th middle phalanx, 5th distal phalanx, and thumb phalanges were short and thick. The first metatarsal, proximal metatarsal, 2nd–5th proximal metatarsal, middle metatarsal, and distal phalanges of both feet were short and thick, and some joint spaces were narrow (Figure 2). The patient was clinically diagnosed with PHP and prescribed 0.25 μg calcitriol, 600 mg vitamin D calcium chewable tablets every 12 h, and 400 IU vitamin D2 soft capsules orally every day.

**TABLE 1:** Initial laboratory examination data of the patient and her younger daughter

Test items	Proband	Youngest daughter	Reference range
Parathyroid (pmol/L)	15.41	70.35	1.60–6.90
Calcitonin (pg/ml)	13.88	/	0.00–6.40
Thyrotropin (μIU/ml)	4.54	5.06	0.35–4.94
Thyroglobulin antibody (IU/ml)	1.35	2.23	0.00–4.11
Thyroid peroxidase antibody (IU/ml)	13.58	19.24	0.00–34.00
Growth hormone (ng/ml)	0.19	6.80	0.13–9.88
Adrenocorticotropic hormone (pg/ml)	92.20	/	7.20–63.30
Cortisol (nmol/L)	534.09	/	171.00–536.00
Alkaline phosphatase (U/L)	/	211.00	43.00–136.00 (^a^143.00–406.00)
Red blood cell count (10^12^/L)	3.17	4.20	3.80–5.10
Hemoglobin (g/L)	96.00	115.00	110.00–150.00
Hematocrit (%)	29.70	37.40	35.00–45.00
Blood potassium (mmol/L)	2.91	3.81	3.50–5.50
Blood sodium (mmol/L)	134.0	138.0	136.0–146.0
Blood calcium (mmol/L)	2.04	1.94	2.10–2.80
Blood magnesium (mmol/L)	0.60	0.88	0.66–1.07
Blood phosphorus (mmol/L)	1.57	1.79	0.80–1.65
Urinary potassium (mmol/24 h)	20.24	25.37	25–125
Urinary sodium (mmol/24 h)	122.3	117.2	130–260
Urinary calcium (mmol/24 h)	1.05	0.17	2.7–7.5
Urinary magnesium (mmol/24 h)	1.19	1.12	1.8–2.4
Urinary phosphorus (mmol/24 h)	5.60	5.04	9.7–42.0
25‐hydroxyvitamin D3 (ng/ml)	21	4.3	20–80
Osteogenic alkaline phosphatase (μg/L)	22.39	/	≤14.30

*Note*: /, No data.

^a^ Child reference range.

The patient's younger daughter visited the hospital because of involuntary hand and foot spasms, and the age at initial diagnosis was 7 years 9 months. Physical examination upon admission revealed a round face and short fingers. Her height, weight, and BMI were 107.6 cm (−3 *SD*), 18 kg (−1.5 *SD*), and 15.5 kg/m^2^, respectively. Laboratory examinations showed considerably increased parathyroid hormone, blood phosphorus, and TSH levels and decreased blood calcium levels. No abnormalities were found on head CT examination. Radiographs of both hands in the right position showed that an ossification center of the distal radius had appeared, and the epiphyseal line was not closed. Seven ossification centers in the wrist and dense white lines in the scaphoid were observed. The metacarpophalangeal epiphysis was partially closed, and the bone age was estimated to be 6–7 years old.

For treatment, 300 mg vitamin D calcium chewable tablets, 400 IU vitamin D2 soft capsules, 0.25 μg alfacalcidol soft capsules, and 12.5 μg levothyroxine sodium were administered orally every day. The patient's younger daughter was followed up regularly at our hospital to adjust the treatment dosage. After 6 months, the PTH level decreased but was still higher than the normal range, the blood calcium level remained normal, and the blood phosphorus level decreased. The height, weight, and estimated growth rate of the younger daughter were 109.6 cm, 18.0 kg, and 3.99 cm/year, respectively.

The patient's mother had short fingers and a height of 155 cm, with no round face or obesity, obvious limb numbness, or history of hand–foot twitching symptoms. The patient's husband and older daughter were in good health and without similar symptoms. The patient and her husband had a non‐consanguineous marriage.

### 3.2 Genetic testing

The results of MS‐MLPA copy number detection in the family indicated that the proband, her mother, and younger daughter had a loss of heterozygosity in the *GNAS‐AS1/GNAS* region, while her husband and older daughter had no abnormalities. Results of the methylation test showed that the proband, her mother, and her younger daughter had abnormal methylation, while her husband and older daughter had no abnormalities. The proband and her younger daughter had *GNAS‐AS1:TSS‐DMR*, *GNAS‐XL:Ex1‐DMR*, and *GNAS A/B:TSS‐DMR* maternal loss‐of‐methylation, with *GNAS‐NESP:TSS‐DMR* gain‐of‐methylation. However, the proband's mother had *GNAS‐AS1:TSS‐DMR*, *GNAS‐XL:Ex1‐DMR*, and *GNAS A/B:TSS‐DMR* gain‐of‐methylation, with *GNAS‐NESP:TSS‐DMR* loss‐of‐methylation. Although we could not obtain the genetic data of proband's grandparents, we speculated that the mutations may have been caused by paternal inheritance (Figure 2a).

WGS produced approximately 150 GB of raw data. The average sequencing depth of the target area was 38.8X, and the coverage of those with an average sequencing depth greater than 10X was approximately 98.8%. Screened CNVs greater than 100 kb are listed in Table 2. The results indicated the presence of seq[GRCh37]del(20)(q13.32) chr20:g.57267263_58117079del in the genome, and the deletion range of the fragment was approximately 849.81 kb (Figure 2b).

**TABLE 2:** Data of the proband with copy number variations (CNVs) ≥100 kb

No.	CNVs	Size (bp)	Gene	OMIM gene
1	seq[GRCh37]dup(X)(q26.1q26.2) chrX:g.130285657_131185430dup	899,774	*FIRRE|IGSF1|OR13H1|STK26*	*IGSF1*
2	seq[GRCh37]del(1)(p21.1p21.1) chr1:g.104139016_104314102del	175,087	*AMY1A|AMY1B|AMY1C|AMY2A*	/
3	seq[GRCh37]dup(2)(p11.2p11.2) chr2:g.87268265_88120780dup	852,516	*CYTOR|LOC285074|LOC730268|MIR4435‐1|MIR4435‐2|MIR4771‐1|MIR4771‐2|PLGLB1|PLGLB2|RGPD1|RGPD2*	/
4	seq[GRCh37]dup(5)(q12.1q12.1) chr5:g.59731982_59932008dup	200,027	*DEPDC1B|PART1|PDE4D*	*PDE4D*
5	seq[GRCh37]del(8)(p23.1p23.1) chr8:g.11942109_12491821del	549,713	*DEFB109A|DEFB130A|DEFB130B|FAM66A|FAM66D|FAM85A|FAM86B1|FAM86B2|FAM90A25P|FAM90A2P|LOC100506990|LOC392196|LOC649352|LOC729732|USP17L2|USP17L7|ZNF705D*	/
6	seq[GRCh37]del(8)(p11.22p11.22) chr8:g.39239317_39389303del	149,987	*ADAM3A|ADAM5*	/
7	seq[GRCh37]del(14)(q11.2q11.2) chr14:g.19424949_20474825del	1,049,877	*BMS1P17|BMS1P18|BMS1P22|DUXAP10|DUXAP9|LINC01297|LINC01297‐DUXAP10‐NBEAP6|LNCRNAATB|LOC100508046|LOC101929572|OR11H2|OR4K1|OR4K15|OR4K2|OR4K3|OR4K5|OR4M1|OR4N2|OR4Q2|OR4Q3|POTEG|POTEH‐AS1|POTEM*	/
8	seq[GRCh37]dup(17)(q21.31q21.31) chr17:g.44404313_44729429dup	325,117	*ARL17A|ARL17B|LRRC37A|LRRC37A2|NSF|NSFP1*	/
9	seq[GRCh37]del(20)(q13.32) chr20:g.57267263_58117079del	849,816	*NPEPL1|PIEZO1P2|MIR296|MIR298|GNAS‐AS1|GNAS|NELFCD|CTSZ|TUBB1|ATP5F1E|PRELID3B|MRPS16P2|ZNF831|EDN3|PIEZO1P1*	*GNAS‐AS1*|*GNAS*|*TUBB1*|*ATP5F1E*|*EDN3*

## 4 DISCUSSION

We report a case of PHP in a Chinese family. The proband and her younger daughter showed clinical manifestations of PTH resistance and AHO. Some distinct features were round face, obesity, and short stature, especially in the proband. In addition, the 25‐hydroxyvitamin D3 of the younger daughter was lower than normal values, which may have contributed to the elevated PTH level, suggesting a risk for osteoporosis occurrence (Mantovani et al., 2018). The mother of the proband had short fingers but showed no PTH resistance, round face, or signs of obesity. The MS‐MLPA test results revealed that the proband and her younger daughter had maternal loss‐of‐methylation in *GNAS‐AS1:TSS‐DMR*, *GNAS‐XL:Ex1‐DMR*, and *GNAS A/B:TSS‐DMR*. The MLPA copy number test indicated that the proband, her younger daughter, and mother had a deletion of the *GNAS‐AS1/GNAS* region fragments. WGS detection showed that the deleted fragment was approximately 849.8 kb, and it contained five pathological genes included in the OMIM database: *GNAS‐AS1*, *GNAS*, *TUBB1*, *ATP5F1E*, and *EDN3*. Based on the clinical manifestations of the proband and her younger daughter, the PHP phenotype, caused by the haploid loss of *GNAS*, was highly matched. We finally diagnosed the patient and her younger daughter with PHP1A and the patient's mother with PPHP.

The manifestations and severity of PHP and related diseases vary among affected individuals. Considerable clinical differences can be found between different types, but the main clinical features include PTH resistance, heterotopic ossification, short finger deformity, and early obesity. The main pathogenesis of PHP is defects in *GNAS*. Gsa, encoded by *GNAS*, can generate heterotrimers with multiple peptide chain transmembrane segments. Activated Gsa can activate adenylate cyclase (AC), catalyze and generate a large amount of cyclic adenosine phosphate, and combine with and activate protein kinase A, thereby catalyzing the phosphorylation of various protein substrates and regulating cell metabolism and gene expression (Mantovani et al., 2018). Therefore, the reduced or inactivated function of Gsa interferes with the downstream AC system and shows various physiological effects, such as resistance/insensitivity to different hormones (Mantovani, 2011). *GNAS* has complex imprinted gene sites with multiple transcription units (Levine, 2012) and is maternally expressed in specific tissues (such as thyroid, gonad, and pituitary gland). Thus, maternal genetic mutations or de novo mutations on the maternal allele can considerably reduce Gsa level or function, causing peptide hormone resistance in conjugation with multiple GPCR signaling pathways, including PTH (Bastepe, 2013).

PHP1A (the most common subtype of PHP) is mainly caused by protein inactivation due to maternal *GNAS* mutations. However, the occurrence of PHP1B is related to the abnormal epigenetic regulation of *GNAS* with no base mutations. Abnormal methylation of the *GNAS A/B:TSS‐DMR* locus can be detected in almost all patients with PHP1B (Mantovani et al., 2018). PHP1C is mainly caused by mutations in exon 13 of *GNAS*. Types 1C and 1A also exhibit multiple hormone resistance and AHO phenotypes, but the difference is that Gsa protein activity in type 1C is normal (Yang et al., 2020). In this study, the patient's 20q13.32 had a deletion of approximately 849 kb. MS‐MLPA analysis suggested that the patient and her younger daughter had maternal deletion of *GNAS* methylation, which led to the occurrence of PHP1A. The clinical manifestation in the patient's mother was PPHP, which may have been caused by a paternal allele mutation. This finding suggests that given the large phenotypic differences observed between individuals, the clinical and molecular diagnosis of familial cases of PHP should include family members, and an in‐depth analysis of genetic mutation characteristics should be conducted.

The correlation between the PHP phenotype and genotype remains unclear, and the disease phenotypes caused by different genotypes overlap widely. The main types of *GNAS* mutations are frameshift, missense, and nonsense (Elli et al., 2018). Snanoudj et al. (2020) conducted a retrospective study of 361 patients with *GNAS* defects in 204 families. They found that approximately 59% were inherited via maternal mutations, and the proportion of their offspring in female patients with PHP1A was as high as 64.7%. However, the deletion of the 20q13.32 segment, including *GNAS*, may result in more diverse clinical phenotypes. To date, only eight cases of relevant CNVs have been reported, with a maximum deletion length of approximately 7.3 Mb and minimum deletion length of approximately 0.7 Mb. Five of these cases were confirmed to have paternal inheritance, one had a de novo mutation, and one had maternal inheritance. Almost all of these patients had low birth weight, feeding difficulties, and mental and growth retardation.

Moreover, the facial features of a broad forehead, triangular face, and sparse eyebrows were common. More than half of the patients (4/7) met the Netchine–Harbison clinical scoring system criteria and were diagnosed with Silver–Russell syndrome (SRS) (Aldred et al., 2002; Balasubramanian et al., 2015; Butler et al., 2013; Geneviève et al., 2005; Liu et al., 2022; Solomon et al., 2011). The phenotype in this study is remarkably different from those previously reported for 20q13.32 deletion mutation, which may have typical PHP characteristics because the deletion of the 20q13.32 segment destroys the maternal Gsa protein activity. However, the paternal 20q13.32 deletion may be more prone to PPHP and SRS‐like phenotypes, such as partial AHO phenotype, growth retardation, and feeding difficulties. Since the number of 20q13.32 deletion mutation reports is rarer than that of *GNAS* SNP/indel, it is necessary to enrich the case reports of maternal 20q13.32 deletion mutation to clarify the correlation between CNVs and phenotype.

In conclusion, we conducted phenotypic and molecular assessments of three patients with PHP from a Chinese Han family. The proband and her younger daughter showed hormone resistance and AHO characteristics, whereas her mother showed a partial AHO phenotype. MS‐MLPA was used to identify maternal loss‐of‐methylation in *GNAS*. WGS further identified that the 20q13.32 fragment had an 849.81 kb deletion. In this study, the phenotypes of the cases were somewhat different from those previously reported in 20q13.32 deletion cases, which may be related to parental imprinting inheritance. Establishing a correct molecular diagnosis process for PHP may be challenging because PHP is caused by different and complex genetic and epigenetic defects.

## AUTHOR CONTRIBUTIONS

Study concepts and design: Y.F., L.L., and L.H. Clinical information collection: L.L., L.W., O.W., and X.X. Data acquisition: O.W., X.X., and A.L. Data analysis/interpretation: Y.F., O.W., and X.X. Manuscript preparation: Y.F. and L.L. Manuscript editing/revision/review: Y.F., X.X., and A.L. All authors confirmed the content of the manuscript.

## CONFLICT OF INTEREST

The authors declare that the research was conducted in the absence of any commercial or financial relationships that could be construed as a potential conflict of interest.

## ETHICS APPROVAL AND CONSENT TO PARTICIPATE

The study was approved by the ethics committee of the Meishan Municipal People's Hospital. Written informed consent was provided by the participant.

## Data Availability

The data supporting the findings of this study are available from the corresponding author upon reasonable request.
